# Retraction Note: The Na/K-ATPase oxidant amplification loop regulates aging

**DOI:** 10.1038/s41598-022-13456-y

**Published:** 2022-06-01

**Authors:** Komal Sodhi, Alexandra Nichols, Amrita Mallick, Rebecca L. Klug, Jiang Liu, Xiaoliang Wang, Krithika Srikanthan, Perrine Goguet-Rubio, Athar Nawab, Rebecca Pratt, Megan N. Lilly, Juan R. Sanabria, Zijian Xie, Nader G.Abraham, Joseph I. Shapiro

**Affiliations:** 1 Departments of Medicine, Surgery, and Biomedical sciences, Joan C. Edwards School of Medicine, Marshall University, Huntington, USA; 2 Department of Medicine and Pharmacology, New York Medical College, Valhalla, NY 10595 USA

Retraction of: *Scientific Reports* 10.1038/s41598-018-26768-9, published online 26 June 2018

The Editors have retracted this Article. After publication, concerns were raised regarding high similarity among the following:

- multiple lanes within each of the Coomassie blue staining images in Figs. 3h, 6c, S1a and S2a;
- DNP blot images in Figs. 3h (H2O2 + pNaKtide group), S1a (both lanes in Y group and left lane in O + WD + P group) and S2a (OB and O groups);
- pSRC blot images in Figs. S2b (OB group) and 3i (pNaKtide, H2O2 and H2O2 + pNaKtide groups).

Further investigation has found that there are unexplained similarities between images representing different groups in:

- Figs. 3a (Control and H2O2 + pNaKtide) and S4a (Control and UV + pNaKtide);
- Fig. S3a (H2O2 + pNaKtide 1 μM and 4 μM, rotated 180 degrees);
- Figs. S3a (H2O2 200 μM) and Fig. S5a (Ouabain 1 nM).

The Editors therefore no longer have confidence in the presented data.

Juan R. Sanabria and Nader G. Abraham agree to this retraction. Komal Sodhi, Jiang Liu, Rebecca Pratt, and Joseph I. Shapiro do not agree to this retraction. Alexandra Nichols, Amrita Mallick, Rebecca L. Klug, Xiaoliang Wang, Krithika Srikanthan, Perrine Goguet-Rubio, Athar Nawab, Megan N. Lilly, and Zijian Xie have not responded to any correspondence from the Editors about this retraction.

